# Increase in Non-AIDS Related Conditions as Causes of Death among HIV-Infected Individuals in the HAART Era in Brazil

**DOI:** 10.1371/journal.pone.0001531

**Published:** 2008-01-30

**Authors:** Antonio G. Pacheco, Suely H. Tuboi, José C. Faulhaber, Lee H. Harrison, Mauro Schechter

**Affiliations:** 1 Department of Epidemiology and Quantitative Methods in Health, National School of Public Health/Scientific Computing Program, Oswaldo Cruz Foundation (FIOCRUZ), Rio de Janeiro, Brazil; 2 Infectious Diseases Epidemiology Research Unit, Graduate School of Public Health and School of Medicine, University of Pittsburgh, Pennsylvania, United States of America; 3 Projeto Praça Onze, Hospital Escola São Francisco de Assis, Universidade Federal do Rio de Janeiro, Rio de Janeiro, Brazil; 4 AIDS Research Laboratory, Hospital Universitario Clementino Fraga Filho, Universidade Federal do Rio de Janeiro, Rio de Janeiro, Brazil; Center for Tobacco Control Research and Education, United States of America

## Abstract

**Background:**

In 1996, Brazil became the first developing country to provide free and universal access to HAART. Although a decrease in overall mortality has been documented, there are no published data on the impact of HAART on causes of death among HIV-infected individuals in Brazil. We assessed temporal trends of mortality due to cardiovascular diseases (CVD), diabetes mellitus (DM) and other conditions generally not associated with HIV-infection among persons with and without HIV infection in Brazil between 1999 and 2004.

**Methodology/Principal Findings:**

Odds ratios were used to compare causes of death in individuals who had HIV/AIDS listed on any field of the death certificate with those who did not. Logistic regression models were fitted with generalized estimating equations to account for spatial correlation; co-variables were added to the models to control for potential confounding. Of 5,856,056 deaths reported in Brazil between 1999 and 2004 67,249 (1.15%) had HIV/AIDS listed on the death certificate and non-HIV-related conditions were listed on 16.3% in 1999, increasing to 24.1% by 2004 (p<0.001). The adjusted average yearly increases were 8% and 0.8% for CVD (p<0.001), and 12% and 2.8% for DM (p<0.001), for those who had and did not have HIV/AIDS listed on the death certificate, respectively. Similar results were found for these conditions as underlying causes of death.

**Conclusions/Significance:**

In Brazil between 1999 and 2004 conditions usually considered not to be related to HIV-infection appeared to become more likely causes of death over time than reported causes of death among individuals who had HIV/AIDS listed on the death certificate than in those who did not. This observation has important programmatic implications for developing countries that are scaling-up access to antiretroviral therapy.

## Introduction

The introduction and widespread use of highly active antiretroviral therapy (HAART) has had a profound impact on the HIV/AIDS epidemic, turning a fatal disease into a manageable chronic condition. As a consequence, marked decreases in AIDS-related morbidity and mortality have been reported in both low- and high-income settings [1]–[5]. However, concerns have been raised about the consequences of prolonged exposure to antiretroviral drugs, with some evidence for an association between the use of protease inhibitors (PIs) and diabetes mellitus (DM) [6] and cardiovascular diseases (CVD) [7], [8]. It has also been recently suggested that HIV itself may play a contributing role in the pathogenesis of some these conditions [9]. Several recent studies conducted in developed countries have documented changes in mortality patterns after the introduction of HAART. Although causes of death traditionally associated with HIV/AIDS continue to play a predominant role, other conditions, including DM, CVD, cancer, liver and renal diseases have been increasingly reported [10]–[16].

In 1996, Brazil became the first developing country to provide free and universal access to HAART. In 2004 147,500 patients were receiving HAART through the Brazil public health system [17]. In 2005 WHO estimated that ART coverage in Brazil was in excess of 80% [18]. Although a decrease in overall mortality has been documented [3], [4], there are no published data on the impact of HAART on causes of death among HIV-infected individuals in Brazil.

To our knowledge, there are no population-based studies that have investigated temporal changes in causes of death among HIV/AIDS patients in developing countries after the introduction of HAART. In the present study, we assessed temporal trends of overall mortality and of selected conditions usually considered not to be related to HIV-infection as causes of death between 1999 and 2004 in individuals who had and who did not have HIV/AIDS listed on their death certificate.

## Results

**Figure 1:**
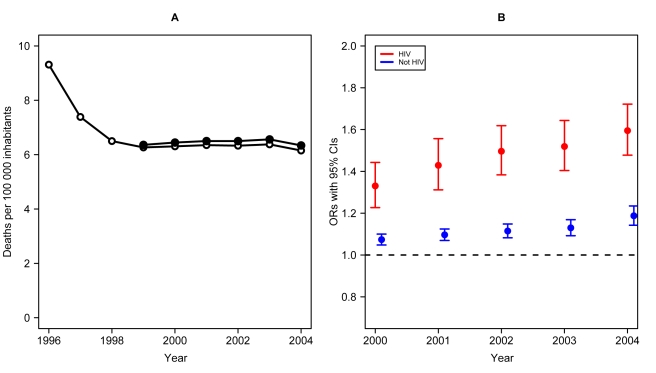
Death Rates and non-HIV-related causes of death. A–Death rates per 100 000 inhabitants of individuals that had HIV/AIDS listed on the death certificate, 1999–2004 (p-value for trend = 0.67, solid circles) and AIDS mortality as an underlying disease from 1996 to 2004 (open circles). B–Odds ratios and 95% confidence intervals of non-HIV-related causes of death listed on the death certificate in individuals who had and who did not have HIV listed on the death certificate. Slopes of trends are significantly different between the two groups (p-value<0.001)

A total of 5,856,056 deaths were reported in Brazil between 1999 and 2004. Of these, 67,249 (1.15%) had HIV/AIDS reported in any field of the death certificate, corresponding to a stable rate of approximately 6.4 cases/100,000 inhabitants per year during the study period (p-value for trend = 0.67, Figure 1A, solid circles). According to official figures from the Brazil Ministry of Health [19], the death rate associated with HIV/AIDS decreased from 9.3/100,000 in 1996 to 6.26/100,000 in 1999, and, according to our data, it has remained relatively stable until 2004 (Figure 1A, open circles).

In the HIV-group, non-HIV-associated causes of death were listed in 16.3% of the death certificates in 1999 and steadily increased to 24.1% in 2004, whereas in the non-HIV group this ranged from 67.4% to 72.1% in the same period. After adjustment for age, gender and state of residence, the average yearly increase of non-HIV-associated causes of death in the HIV group was 7.98% (95%CI = 6.65,9.33; p-value<0.001) and 2.98% for the non-HIV group (95%CI = 2.22,3.75; p-value<0.001). The slopes of the increase for the two groups were significantly different (p-value<0.001). Figure 1B shows temporal trends for the HIV and for the non-HIV groups, with year as a categorical variable, setting 1999 as the baseline year. In this model, the OR for having a non-HIV-associated condition listed on the death certificate in 2004 compared to 1999 was 1.6 (95%CI = 1.48,1.72; p-value<0.001) for the HIV group, and 1.19 (95%CI = 1.14,1.23; p-value<0.001) for the non-HIV group and (Table 1); the interaction with year as a categorical variable was also highly significant (p-value<0.001).

**Table 1:** Description and odds ratios of having a non-HIV related condition* in any field of the death certificate stratified by reported HIV status**

Variable		non-HIV (N = 5788807)			HIV (N = 67249)		
		n (%)	OR (95% CI)	P-value	n (%)	OR (95% CI)	P-value
Outcome	Non-HIV-related	4059422 (70.12%)			14746 (21.93%)	-	-
**Year of death**	**1999**	625767 (67.43%)	Reference		1774 (16.6%)	Reference	
	**2000**	649305 (69.39%)	1.07 (1.05–1.1)	<0.001	2317 (21.09%)	1.33 (1.23–1.44)	<0.001
	**2001**	665383 (70.02%)	1.1 (1.07–1.12)		2494 (22.24%)	1.43 (1.31–1.56)	
	**2002**	685628 (70.58%)	1.11 (1.08–1.15)		2628 (23.11%)	1.5 (1.38–1.62)	
	**2003**	702692 (70.93%)	1.13 (1.09–1.17)		2737 (23.56%)	1.52 (1.4–1.64)	
	**2004**	730647 (72.15%)	1.19 (1.14–1.23)		2796 (24.58%)	1.6 (1.48–1.72)	
**Gender**	**Female**	1659581 (68.4%)	Reference		4369 (20.98%)	Reference	
	**Male**	2399841 (71.37%)	1.08 (1–1.17)	0.015	10377 (22.35%)	1.06 (1.01–1.11)	0.03
**Age group**	**<15**	97573 (19.46%)	Reference		232 (14.14%)	Reference	
	**15–29**	352801 (83.44%)	20.44 (16.83–24.81)	<0.001	2057 (15.9%)	1.15 (1–1.33)	<0.001
	**30–39**	262805 (76.66%)	12.99 (11.2–15.07)		5387 (20.39%)	1.5 (1.31–1.71)	
	**40–49**	390424 (76.88%)	12.79 (11.14–14.69)		4186 (24.59%)	1.89 (1.65–2.16)	
	**50–59**	535089 (78.34%)	13.85 (12.1–15.86)		1881 (29.81%)	2.47 (2.14–2.86)	
	**60+**	2400943 (72.69%)	10.39 (9.07–11.9)		974 (35.63%)	3.25 (2.81–3.76)	
	**Unknown**	19787 (71.11%)	9.29 (7.93–10.88)		29 (15.85%)	1.17 (0.76–1.8)	

* non-HIV-related neoplasms (C00-C80, except C46–Kaposi's sarcoma), DM (E10-E14), CVD (I00-I99), except cardiac arrest (I46), digestive diseases (K00-K93), genital-urinary diseases, (N00-N99) and external causes (S00-Y98)

** Adjusted for state of residency

**Figure 2:**
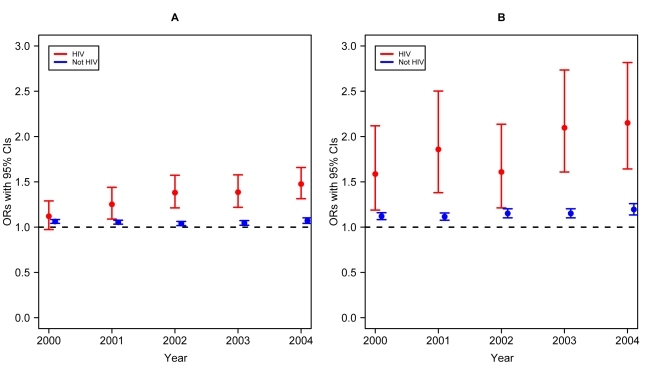
CVD and DM in HIV-infected/AIDS patients. Odds ratios and 95% confidence intervals comparing the chance of having the disease listed on the death certificate over time, compared with 1999. A–CVD; B–DM. Slopes for trends are significantly different between groups for CVD (p-value<0.001) and DM (p-value<0.001).

CVD increased in the HIV group from 4.3% in 1999 to 6.4% in 2004 (adjusted average increase of 7.79% per year; (95%CI = 5.74,9.66; p-value<0.001). In the non-HIV group, an increase of 0.80% per year was observed (95%CI = 0.28,1.33; p-value = 0.002), from 36.2% in 1999 to 39.3% in 2004. Compared to 1999, the OR for having CVD listed on the death certificate in 2004 was 1.5 (95%CI = 1.34,1.68; p-value<0.001) for the HIV group and 1.07 (95%CI = 1.04,1.10; p-value<0.001) for the non-HIV groups (Table 2). Temporal trends for both groups are shown in Figure 2A; both interactions between year as a continuous or a categorical variable were significantly different between the two groups (p-value<0.001).

**Table 2:** Description and odds ratios of having CVD* in any field of the death certificate stratified by reported HIV status**

Variable		non-HIV (N = 5788807)			HIV (N = 67249)		
		n (%)	OR (95% CI)	P-value	n (%)	OR (95% CI)	P-value
Outcome	CVD	2196228 (37.94%)			3746 (5.57%)	-	-
**Year of death**	**1999**	341452 (36.8%)	Reference		491 (4.59%)	Reference	
	**2000**	360784 (38.56%)	1.07 (1.05–1.09)	<0.001	579 (5.27%)	1.14 (1–1.3)	<0.001
	**2001**	368185 (38.75%)	1.06 (1.04–1.09)		647 (5.77%)	1.25 (1.1–1.43)	
	**2002**	375371 (38.64%)	1.05 (1.02–1.07)		721 (6.34%)	1.38 (1.22–1.57)	
	**2003**	386117 (38.97%)	1.05 (1.02–1.07)		750 (6.46%)	1.39 (1.23–1.57)	
	**2004**	404072 (39.9%)	1.07 (1.04–1.1)		800 (7.03%)	1.5 (1.34–1.68)	
**Gender**	**Female**	1067296 (43.99%)	Reference		1241 (5.96%)	Reference	
	**Male**	1168685 (34.76%)	0.79 (0.75–0.82)	<0.01	2747 (5.92%)	0.98 (0.91–1.05)	0.96
**Age group**	**<15**	23306 (4.65%)	Reference		101 (6.15%)	Reference	
	**15–29**	33154 (7.84%)	1.75 (1.43–2.14)	<0.001	480 (3.71%)	0.59 (0.47–0.75)	<0.001
	**30–39**	62250 (18.16%)	4.46 (4.01–4.97)		1331 (5.04%)	0.79 (0.62–0.99)	
	**40–49**	162441 (31.99%)	9.22 (8.54–9.95)		1103 (6.48%)	1.01 (0.8–1.28)	
	**50–59**	289154 (42.33%)	14.33 (13.34–15.39)		590 (9.35%)	1.51 (1.18–1.93)	
	**60+**	1661862 (50.31%)	19.56 (18.18–21.05)		379 (13.86%)	2.37 (1.85–3.04)	
	**Unknown**	3814 (13.71%)	3.53 (2.48–5.02)		4 (2.19%)	0.35 (0.11–1.16)	

* CVD: ICD10 I00-I99, except cardiac arrest (I46)

** Adjusted for state of residency

A similar phenomenon was observed for DM, in which an adjusted annual increase of 12.3% (95%CI = 8.13,16.56;, p-value<0.001), from 0.6% in 1999 to 1.4% in 2004 was observed in the HIV group, in contrast with an adjusted increase of 2.83% per year in the non-HIV group (95%CI = 1.95,3.72,, p-value<0.001), from 6.2% in 1999 to 7.7% in 2004 (Figure 2 B, Table 3). Compared to 1999, the OR for having DM listed on the death certificate in 2004 was 2.16 (95%CI = 1.65,2.82; p-value<0.001) for the HIV group and 1.2 (95%CI = 1.13,1.26; p-value<0.001) for the non-HIV group. The increase in the HIV group was again steeper than in the non-HIV group, both with year as a continuous or as a categorical variable (p<0.001).

**Table 3:** Description and odds ratios of having DM* in any field of the death certificate stratified by reported HIV status**

Variable		non-HIV (N = 5788807)			HIV (N = 67249)		
		n (%)	OR (95% CI)	P-value	n (%)	OR (95% CI)	P-value
Outcome	DM	411862 (7.11%)			744 (1.11%)	-	-
**Year of death**	**1999**	57312 (6.18%)	Reference		63 (0.59%)	Reference	
	**2000**	65525 (7%)	1.12 (1.08–1.16)	<0.001	109 (0.99%)	1.62 (1.21–2.15)	<0.001
	**2001**	66886 (7.04%)	1.12 (1.08–1.16)		130 (1.16%)	1.87 (1.39–2.52)	
	**2002**	71025 (7.31%)	1.15 (1.1–1.2)		117 (1.03%)	1.63 (1.23–2.17)	
	**2003**	72933 (7.36%)	1.15 (1.1–1.2)		161 (1.39%)	2.13 (1.63–2.77)	
	**2004**	78181 (7.72%)	1.2 (1.13–1.26)		164 (1.44%)	2.16 (1.65–2.82)	
**Gender**	**Female**	235268 (9.7%)	Reference		240 (1.15%)	Reference	
	**Male**	176594 (5.25%)	0.59 (0.55–0.64)	<0.01	504 (1.09%)	0.9 (0.77–1.04)	0.19
**Age group**	**<15**	617 (0.12%)	Reference		0 (0%)	Reference	
	**15–29**	3309 (0.78%)	Reference		42 (0.32%)	Reference	
	**30–39**	6811 (1.99%)	4.86 (3.96–5.95)	<0.001	171 (0.65%)	2.15 (1.53–3.03)	<0.001
	**40–49**	22329 (4.4%)	10.59 (8.74–12.82)		244 (1.43%)	4.68 (3.37–6.49)	
	**50–59**	57967 (8.49%)	20.94 (17.32–25.32)		163 (2.58%)	8.58 (6.15–11.97)	
	**60+**	320444 (9.7%)	23.07 (19.04–27.96)		122 (4.46%)	15.24 (10.96–21.19)	
	**Unknown**	385 (1.38%)	3.89 (2.06–7.33)		2 (1.09%)	4 (1–16.09)	

* DM: ICD10 E10-E14, except cardiac arrest (I46)

** Adjusted for state of residency

In the analysis of underlying causes of death among HIV-infected patients, non-HIV-related conditions, CVD and DM increased significantly from 1999 to 2004, a result that corroborates our findings when all causes listed on death certificates were analyzed (data not shown).

## Discussion

The online availability of all death certificates issued in Brazil provided the opportunity to compare reported causes of death in 1999, the first year in which death certificates contained primary, secondary, and contributing causes of death according to ICD-10 codes, with later years among individuals who had HIV/AIDS listed on the death certificate.. To our knowledge, this is the first report on temporal changes in causes of death among HIV/AIDS patients at the population level in a developing country in the HAART era.

The present study suggests that, in Brazil, similar to what has been reported from developed countries, mortality patterns among HIV/AIDS patients are changing in the HAART era. We found that, in comparison to 1999, there was a steady and significantly larger increase in the frequency with which conditions not usually associated with HIV/AIDS were listed as causes of death increased for individuals who also had HIV listed on the death certificate than in individuals who did not, from 16.3% in 1999 to 24.1% in 2004, representing 14,746 deaths in the period. In particular, listing of CVD or DM as causes of death represented 3,746 and 744 deaths, respectively, both appearing to become more likely causes of death over time in individuals whose death certificate also included HIV/AIDS than in those who did not. Additionally, during the same period there were statistically significant increases in these conditions as underlying causes of death among individuals who had HIV/AIDS mentioned on their death certificates.

We speculate that theses changes are not explained by aging of the population alone, given that the mean age of death in the non-HIV group increased marginally more than the mean age of death in the HIV group (data not shown). Thus, certain potentially preventable and/or treatable conditions, such as CVD and DM, may have played significant roles in these changes, given that the proportion of death certificates in which these conditions are listed increased significantly faster in individuals for whom HIV was listed on the death certificate than in those for whom it was not listed.

Our results are in agreement with reports from developed countries where the sharp decrease in mortality following the introduction of HAART was accompanied by significant changes in mortality patterns among HIV-infected individuals. In these countries, after a steep decrease in mortality rates following the introduction of HAART, mortality rates have been reasonably stable since the late 1990's. For example, in the United States, mortality rates declined abruptly in 1994/1995, but remained stable from 1998 onwards, at approximately 7 deaths/100,000 population [20], [21]. In countries where HIV prevalence is well defined and thus could be used as the denominator, a steady increase in the proportion of deaths attributed to conditions that generally are not attributed to HIV infection, such as CVD and DM [5], [10]–[14], has been reported. We were not able to perform similar analyses, given the absence of reliable estimates of HIV prevalence in most regions of Brazil.

Our results are also in agreement with what has been reported in population-based studies conducted in developed countries. For example, combining information on HIV/AIDS surveillance in New York City with vital statistics data, Sackoff et al. [11] showed an increase in the proportion of non-HIV/AIDS-related causes of death from 19.8% to 26.3% between 1999 and 2004, with CVD ranking second overall and DM reaching 4.4% of all non-HIV-related causes of death in women. In another population-based study in the U.S., Selik et al., using a similar approach to ours but limiting the analysis only to those who had HIV/AIDS listed on the death certificate, showed that CVD increased from 4% to 7.7% from 1987 to 1999, with a steeper increase between 1996 and 1999 [14]. Similar trends have also been documented in cohort studies. In the CASCADE Collaboration, CVD and DM as a group increased from 1.3% in the pre-HAART era to 4.3% in post-HAART era [12]. In the HIV Outpatient Study, Palella et al. showed that non-HIV/AIDS-related causes of death increased from 13.1% to 42.5% between 1996 and 2004 in 12 clinics in the United States, with CVD being leading cause of death in this group in 2004 [13]. Crum et al., studying an HIV cohort of U.S. military beneficiaries, also showed increases in cardiac disease (from 8% to 22%) and DM (from 0% to 3%) being listed on death certificates in the pre- and post-HAART era, respectively [10]. In a simulation study, Braithwaite et al predicted that HIV-infected patients will be increasingly dying of co-morbidities not related to HIV infection. For example, they predicted that a 30 years old patient with a high CD4 count and low viral load would have a 45% chance of dying from non-HIV related causes, and that up to 35% of these would be CVD [15]. Given the absence of reliable estimates of HIV prevalence in most regions of Brazil, we were not able to provide estimates of actual trends among HIV-infected individuals. Nonetheless, we believe that our findings are likely to reflect a changing mortality pattern among HIV/AIDS patients in Brazil that could be associated with widespread availability of HAART [22], a pattern similar to that in developed countries. It should be noted that it is estimated that over 80% of HIV infected patients who, according to international guidelines, were in need of treatment in Brazil were on antiretroviral therapy during the study period[18].

A major strength of our study is that we analyzed all available data from all death certificates issued in a large developing country over a six year period, which allowed us to investigate temporal trends in causes of death by comparing individuals who had HIV/AIDS listed in their death certificates to those who did not. Furthermore, the use of any mention of conditions on the death certificate as opposed to only the underlying cause of death allowed us to overcome one of the limitations of the current ICD system, which does not cover some diseases associated with HIV [16], as well as to capture the contributing effect of other conditions. Since HIV-infected individuals now live longer [10], [13] and thus have longer periods of time at risk for chronic conditions associated with aging, an increase in the frequency of CVD and DM is to be expected. There are also data that indicate an association between certain antiretroviral drugs as well as time on therapy and risk for CVD [7], [23]. Additionally, recent data suggest that HIV replication may be associated with increased levels of pro-inflammatory markers which, in turn, are involved in the pathogenesis of CVD [9].

Our study has several limitations. Most importantly, we only analyzed data from death certificates, which may lack sensitivity and specificity for medical conditions [24]–[26]. Nonetheless, by using an approach akin to MOR [27], a strategy that is commonly utilized in studies that investigate occupational hazards, we were able to estimate relative risks by comparing individuals who had HIV/AIDS cited in their death certificates to those who did not. The main difference from the classical use of MOR was that the focus of our analysis was not to compare risks between groups, but to compare temporal trends among them. This approach was chosen due to the lack of reliable estimates of the prevalence of HIV infection in Brazil, particularly age and gender distribution. Additionally, as is the case for all population-based studies, we cannot exclude the influence of unknown confounders that may have contributed to increased reports of non-HIV associated causes of death or to changes over time in the frequency in which HIV/AIDS is listed on death certificates. The latter, if present, probably did not play a significant role, given the stable rate of HIV/AIDS being listed on death certificates, which in turn is in agreement with reports that indicate that HIV-related mortality has remained stable since 1999 [3], [4]. Information on the proportion of those who had HIV/AIDS listed on their death certificate and who were or had been on antiretroviral therapy is not available, as well as other important confounders such as smoking and other risk factors for CVD, and thus could not be assessed in our analyses. However, since HAART is freely and universally available for all those who qualify for treatment according to treatment guidelines that are virtually identical to those used in developed countries [28], [29], it is likely that the vast majority of those who had HIV/AIDS listed on the death certificate were or had been on HAART.

In conclusion, this is the first study to examine changes in mortality patterns among HIV-infected individuals in Brazil after the introduction of HAART. Of particular importance is the finding that conditions that potentially may be prevented and/or treated, such as CVD and DM, have appeared to become more likely causes of death over time among HIV-infected individuals than in the general population. The immediate implication of this finding is that the clinical management of persons living with HIV/AIDS should include prevention, diagnosis, and treatment of chronic conditions, such as CVD and DM. At the program level, the Brazilian network of treatment facilities will need to increase its capacity to diagnose and manage co-morbidities that can influence outcome. This, in turn, reinforces the need to integrate HIV/AIDS programs with other public health programs, in order to establish a healthcare infrastructure that is capable to take the necessary measures to prevent, diagnose, and treat these conditions [30]. Finally, for other developing countries that are just starting to scale-up their programs, lessons learned from the Brazilian experience might help to prepare in advance to changes in morbidity and mortality patterns that will likely occur once HAART becomes widely available.

## Materials and Methods

In Brazil the death certificate is a standardized form that is filled out by a physician. Until 1998, death certificates only included the underlying cause of death. Since 1999, death certificates, besides containing demographic information, include primary, secondary, and contributing causes of death according to the International Classification of Diseases 10th revision (ICD-10) codes [24]. All death certificates issued in Brazil are entered in datasets without personal identifiers and are available online at http://tabnet.datasus.gov.br/tabdata/sim/dados/cid10_indice.htm. This national database is known as the Brazilian Mortality Information System (Sistema de Informações sobre Mortalidade [SIM]).

In this study, we investigated trends for all causes of death mentioned on death certificates in Brazil between 1999 and 2004. During the study period Brazil had a population of approximately 173 million people [31]. We compared temporal trends in causes of death among individuals who had HIV/AIDS listed in any field of the death certificate (ICD-10 codes B20-B24, Z21), referred to as the HIV group, with those who did not have HIV/AIDS reported on the death certificate (the non-HIV group) using an approach akin to the mortality odds-ratio (MOR) focusing on temporal trends, not on the risk of dying with the studied causes, which would be the classical use of MOR [27].

We conducted three separate analyses, in which the outcomes were defined as the presence or absence in any field of the death certificate of: [I] non-HIV-associated causes, defined as non-HIV-related neoplasms (C00-C80, except C46–Kaposi's sarcoma), DM (E10-E14), CVD (I00-I99), except cardiac arrest (I46), digestive diseases (K00-K93), genital-urinary diseases, (N00-N99) and external causes (S00-Y98); [II] CVD (I00-I99), except cardiac arrest (I46); and [III] DM (E10-E14). Additionally, we also examined these outcomes as the underlying cause of death, as defined by the World Health Organization (WHO), in individuals who had HIV/AIDS mentioned on their death certificates [32]. The underlying cause of death, which consists of only one cause per death certificate, is coded by local health departments based on standard rules [33], and is the official figure reported by the Brazil Ministry of Health to WHO for mortality statistics.

Logistic regression models were fitted and co-variables were added to the models to control for possible confounding, including age group (<15; 15–29; 30–39; 40–49; 50–59; > = 60 years); gender; year of death; and state of residence. Age groups were used to avoid low numbers in some regions of the curves. Generalized estimating equations (GEE) were used to fit the logistic models and to account for spatial correlation structures in the data (i.e. using state of residence as clusters), which were assumed to be interchangeable for this analysis.

Year of death was treated either as a continuous (linear) or categorical variable in the models. In the former case, linear trends are reported as percentages per year, while in the latter, odds ratios are used to compare yearly changes to the baseline year of 1999. Differences in slopes in temporal trends were tested by an interaction term between HIV status and year. Reference groups were <15 years for age, female for gender, and São Paulo State for state of residence.

All analyses were performed in R for Windows v. 2.4.1 [34], using the package ‘geepack’ for GEE estimation [35].
